# Acute Kidney Injury Complicating Critical Forms of COVID-19: risk Factors and Prognostic Impact

**DOI:** 10.12688/f1000research.144105.3

**Published:** 2025-01-02

**Authors:** Jihene Guissouma, Hana Ben Ali, Hend Allouche, Insaf Trabelsi, Olfa Hammami, Yosra Yahia, Ghadhoune Hatem

**Affiliations:** 1 Medical intensive care unit of Bizerte University Hospital, Bizerte, 7021, Tunisia; 2 University of Tunis El Manar Faculty of medicine of Tunis, Tunis, 1007, Tunisia; 3 Pediatrics department of Bizerte University Hospital, Bizerte, 7021, Tunisia; 4 Emergency department of Rabta University Hospital, Tunis, 1007, Tunisia

**Keywords:** acute kidney injury, coronavirus disease 2019, mortality, risk factors, prognosis.

## Abstract

**Background:**

Severe acute respiratory syndrome coronavirus-2 (SARS-CoV-2) mainly affects the respiratory tract, but different organs may be involved including the kidney. Data on acute kidney injury (AKI) in critical forms of coronavirus disease 2019 (COVID-19) are scarce. We aimed to assess the incidence, risk factors and prognostic impact of AKI complicating critical forms of COVID-19.

**Methods:**

A retrospective descriptive case/control monocentric study conducted in a medical intensive care unit of a tertiary teaching hospital over a period of 18 months.

**Results:**

We enrolled 144 patients, with a mean age of 58±13 years old and a male predominance (sex-ratio: 1.25). Forty-one (28%) developed AKI within a median of 4 days (Q1: 3, Q3: 8.5) after hospitalization. It was staged KDIGO class 3, in about half of the cases. Thirteen patients underwent renal replacement therapy and renal function improved in seven cases. Diabetes (OR: 6.07; 95% CI: (1,30-28,4); p: 0.022), nephrotoxic antibiotics (OR: 21; 95% CI: (3,2-146); p: 0.002), and shock (OR: 12.21; 95% CI: (2.87-51.85); p: 0.031,) were the three independent risk factors of AKI onset. Mortality was significantly higher in AKI group (HR:12; 95% CI: (5.81-18.18); p:0.041) but AKI didn’t appear to be an independent risk factor of poor outcome. In fact, age > 53 years (p: 0.018), septic shock complicating hospital acquired infection (p: 0.003) and mechanical ventilation (p<0.001) were the three prognostic factors in multivariate analysis.

**Conclusions:**

The incidence of AKI was high in this study and associated to an increased mortality. Diabetes, use of nephrotoxic antibiotics and shock contributed significantly to its occurrence. This underlines the importance of rationalizing antibiotic prescription and providing adequate management of patients with hemodynamic instability in order to prevent consequent AKI.

## Introduction

Since its first outbreak in December 2019 in China, the coronavirus disease 2019 (COVID-19) has spread rapidly all over the world causing a serious pandemic with high morbidity and mortality. Severe acute respiratory syndrome coronavirus-2 (SARS-CoV-2) mainly affects the respiratory tract with a variable clinical presentation ranging from asymptomatic forms to severe pneumonia with acute respiratory distress syndrome (ARDS) and death. ^1^ Although, physicians must be aware of the possible damage of other organs causing a multi-systemic impairment. ^2^ Acute kidney injury (AKI) is a frequent complication in COVID-19 patients with a reported incidence widely ranging from 0.5% ^3^ to above 80%. ^4^^,^^5^

The incidence of AKI increases in parallel with the COVID-19 severity and the highest rates were recorded in the intensive care unit (ICU) patients. In addition, the occurrence of AKI seems to be a poor prognostic factor with an increased mortality. ^6^^,^^7^

Aside the renal tropism of the SARS CoV-2, the pathogenesis of AKI appears to be multifactorial. Different mechanisms have been incriminated, including cells viral invasion via angiotensin converting enzyme 2 receptors mainly present on the proximal tubule cells, imbalance of the renin-angiotensin-aldosterone system, prothrombotic coagulopathy and the release of nephrotoxic mediators from cytokine storm. ^8^ Non-specific mechanisms such as drug nephrotoxicity and renal hypoperfusion also play an important role. ^9^

Currently, several published studies focused on hospitalized patients with COVID-19 and AKI but data on AKI complicating critical forms of COVID-19 from the Great Maghreb and particularly from Tunisia are scarce. In this study we aimed to assess the incidence, the risk factors and the prognostic impact of AKI complicating critical forms of COVID-19.

## Methods

### Design

This was a retrospective descriptive case/control monocentric study carried out in the medical ICU of Bizerte hospital (a tertiary teaching hospital in north of Tunisia) over a period of 18 months (September 2020-February 2022). This medical ICU is managed by medical intensivists with a novel unit of six beds created for the COVID-19 outbreak.

### Study endpoints

The primary endpoint was the incidence of AKI complicating critical forms of COVID-19. The second endpoint was ICU mortality.

### Patients

All adult patients (>18 years) admitted to the ICU for critical forms of COVID-19 during the study period were included. Patients with a history of chronic kidney disease (CKD) were excluded in order to have a homogeneous group and avoid confounding factors. Those who did not meet the critical COVID-19 criteria were also excluded. Laboratory-confirmation of COVID-19 diagnosis was performed by detection of the SARS-CoV-2 RNA in nasal swabs using reverse transcription-polymerase chain reaction. Patients were divided in two groups: the case group which included the critical COVID-19 patients who developed AKI during their ICU stay according to the Kidney Disease: Improving Global Outcomes (KDIGO) classification: AKI patients, and the control group which included those who didn’t develop AKI during their ICU stay according to the same classification: No AKI patients.

All patients included had at least one creatinine measurement on ICU admission and one or more prior measurement in the department from which they were transferred.

### Definitions

- Critical form of COVID-19 was considered in all included patients as defined by the WHO: “criteria for acute respiratory distress syndrome (ARDS), sepsis, septic shock, or other conditions that would normally require the provision of life-sustaining therapies such as mechanical ventilation (invasive or non-invasive) or vasopressor therapy”. ^1^
- Sepsis was defined according to the 3rd international consensus (Sepsis-3): “presence of organ dysfunction (identified as an acute change in total Sequential Organ Failure Assessment [SOFA] score ≥2 points), consequent to the infection”. ^10^ Only sepsis prior to AKI development was assessed as a risk factor when no other cause has been found. AKI was related to sepsis when a new episode of sepsis occurred during hospitalization and was followed within 48 hours by AKI.
- AKI was defined by the Kidney Disease: Improving Global Outcomes (KDIGO) as any of the following: increase in serum creatinine (SCr) by ≥0.3 mg/dl (26.5 μmol/L) within 48 h; or ≥1.5 times baseline (within the prior seven days) or urine volume < 0.5 ml/Kg/h for six hours. AKI was staged for severity according to the KDIGO criteria. Stage 1 involves increase in SCr to 1.5–1.9 times baseline or ≥ 0.3 mg/dl (26.5 μmol/L) and/or urine output <0.5 ml/kg/hr for 6–12 hours. Stage 2 is considered when SCr increases to 2.0–2.9 times baseline and/or urine output <0.5 ml/kg/hr for >12 hours. Stage 3 is defined by increase in SCr to 3.0 times baseline, or to >4.0 mg/dl (353.6 μmol/L), initiation of renal replacement therapy (RRT), and/or urine output <0.3 ml/kg/hr for ≥24 hours, or anuria for ≥12 hours. ^11^
- For patients who had previous creatinine measurement in the 7-365 days prior to admission, the most recent value was taken as the baseline creatinine ^12^ and for whom no prior value was available, the lowest creatinine measured in the original department before transfer to ICU was considered as the baseline creatinine.
- Full renal recovery was achieved when serum creatinine reached a value below 1.5 times baseline and urine volume >0.5 ml/kg/h. ^12^
- Rhabdomyolysis was retained if the creatine phosphokinase (CPK) rate was greater than five times the upper limit of normal. ^13^ Normal CPK rates range from 10 to 200 UI/L according to our hospital laboratory.
- The most prescribed nephrotoxic drugs in our ICU are vancomycin, aminoglycosides and colistin.
- Omnipaque 300 (Tunisian Central Pharmacy code = 507659) was the iodine contrast agent used in our hospital.

### Therapeutic management

- Oxygen support was: noninvasive including noninvasive ventilation (NIV) and high-flow nasal cannula (HFNC); or invasive for patients requiring mechanical ventilation (MV).
- Prone position was indicated for awake and coopering patients or those under MV having PaO2/FiO2 < 150.
- Corticosteroids (dexamethasone 6 mg/day; Tunisian Central Pharmacy code = 350366), vitamin C supplementation (Tunisian Central Pharmacy code = 352910), and anticoagulation were also prescribed. Our ICU anticoagulation protocol was based on low molecular weight heparin (LMWH). Standard prophylactic dose (enoxaparin 0.4 ml/day; Tunisian Central Pharmacy code = 352177) was prescribed to patients with body mass index (BMI) <30 kg/m ^2^ and intermediate dose (enoxaparin 0.4 ml ×2/day) for those with BMI ≥30 kg/m ^2^. Patients with presumed or confirmed venous thromboembolism had curative anticoagulation with enoxaparin 100 UI/kg×2/day. After the onset of AKI and in cases of creatinine clearance < 30 ml/min LMWH was switched to calciparin (Tunisian Central Pharmacy code = 505612) or unfractionated heparin (Tunisian Central Pharmacy code = 353526).
- Antibiotics were prescribed when bacterial co-infection was presumed or confirmed. All these drugs were supplied by our hospital internal pharmacy.

### Assessed data

We focused for each patient on demographic (age and gender) and clinical features (comorbidities, initial pleuropulmonary, cardiovascular and neurological examinations data), initial laboratory findings (arterial blood gases, renal function tests, complete blood count, CRP levels, prothrombin time and CPK), initial thoracic computed tomography (CT) scan data, drugs received prior to AKI onset, respiratory support, renal function during hospitalization, need for RRT, ICU length of stay (LOS) and mortality.

The classification of the French “Société d’Imagerie Thoracique” was used to assess lesions extension. It’s based on visual assessment of parenchymal extension. Five stages were considered according to the percentage of lung affected: absent or minimal involvement (<10%), moderate (10-25%), extensive (25-50%), severe (50-75%) or critical (>75%). ^14^

### Statistical analysis

Free open Jamovi software was used for data collection and analysis. ^15^ For the descriptive study, we calculated means with standard deviations for quantitative variables with a Gaussian distribution and medians with interquartile range for variables with a non-Gaussian distribution. These variables were compared with a nonparametric Mann-Whitney test. We calculated counts and percentages for qualitative variables. Percentages were compared with Pearson’s chi-square test and with Fisher’s exact test, if this test was invalid. For analytic study; univariate logistic regression model then multivariate logistic regression analysis was done to assess AKI risk factors. The receiver operating characteristic (ROC) curve was used to ascertain the cut-off values of the continuous data, which were subsequently converted into dichotomous form. A comparison survival curve was obtained by means of the Log Rank test, and a multivariable Cox regression model was employed to identify independent factors associated with mortality.

In all statistical tests, the significance threshold was set at 0.05.

### Ethical considerations

The Ethics Committee of our hospital (Habib Bougatfa hospital of Bizerte Tunisia) approved the study on July 20, 2023 (Approval number 1/2023) and waived informed consent because of the retrospective and descriptive design of the study. The principles outlined in the Declaration of Helsinki were followed in the protocol study.

With the aim of carrying out this work by the end of 2022, we called all surviving patients and relatives of deceased ones who met the inclusion criteria to obtain their consent to use their data anonymously and confidentially. Unfortunately, we were unable to reach all of them. We therefore obtained consent from 31 surviving patients (51 survivors in total) and consent from 44 suitable legal guardians of deceased patients (93 deceased in total). As we were unable to obtain consent from a significant number of the patients we wished to include, we referred this problem to our hospital's ethics committee. As this was a retrospective, observational study, and it was impossible to contact all the patients or their relatives, the ethics committee members waived informed consent for those we could not reach, and we obtained their agreement to carry out this study.

## Results

### Baseline characteristics, therapeutics and evolution

**Figure 1.**
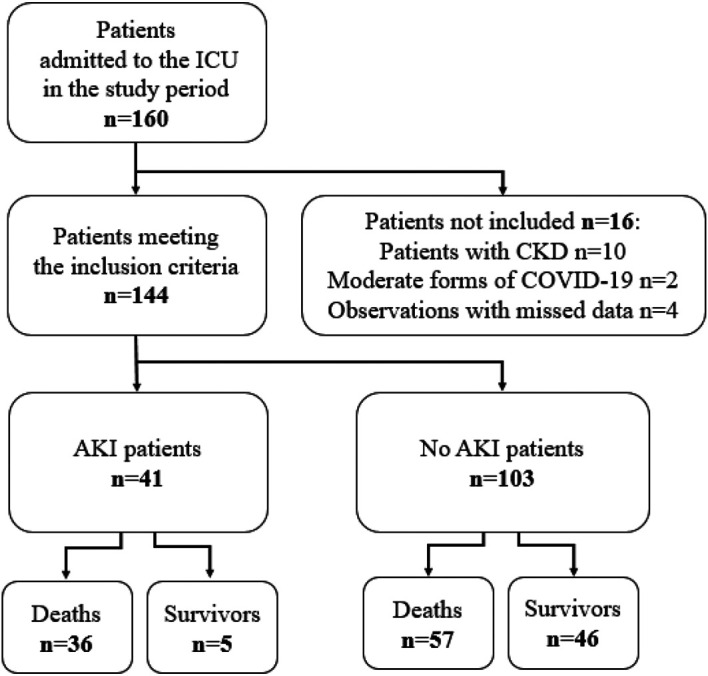
Patient flow chart.

Among 160 patients who were admitted to the ICU in the study period, 16 didn’t meet the inclusion criteria. Thus, overall, 144 patients were included. Seventy-eight (54%) were transferred from COVID units, 42 (29%) from the emergency department and 24 (17%) from other medical or chirurgical units. Forty-one (28%) patients developed AKI ( Figure 1).

Table 1 shows the characteristics and the evolution of all patients and both groups: AKI and No AKI patients. We have summarized the epidemiological and clinical features, in addition to the laboratory and CT scan findings at ICU admission. Predisposing conditions to AKI, therapeutics and evolution were also assessed. In fact, AKI patients were older and had more comorbidities (notably diabetes and hypertension). Their heart rate, mean arterial pressure (MAP) and severity scores on admission were also higher compared to No AKI patients. Initial laboratory findings showed higher levels of white blood cells count (WBC) and C reactive protein (CRP). In addition, their baseline serum urea and creatinine rates on admission were higher. Nephrotoxic antibiotics, shock and MV requirement were the main predisposing conditions to AKI.

**Table 1.** Characteristics and evolution of all patients and both groups according to AKI occurrence.

Variable	Total population (n=144)	AKI patients (n=41)	No-AKI patients (n=103)	OR (95% CI)	P-value
**Age (years)**	58±13	62±11	56±14		**0.005**
**Sex-ratio **	1.25	1.28	1.24		0.934
**BMI**	29±5	28±4	29±5		0.420
**Comorbidities**					
Diabetes	62 (43)	26 (63)	36 (35)	**1.81 (1.27-2.57)**	**0.002**
Hypertension	60 (42)	27 (66)	33 (32)	**2.05 (1.43-2.93)**	**< 0.001**
dyslipidemia	24 (18)	7 (17)	17 (17)		0.969
Cardiomyopathy	20 (14)	3 (7)	17 (16)		0.150
Chronic respiratory failure	5 (3)	3 (7)	2 (2)		0.112
Immunocompromised	4 (3)	2(5)	2 (2)		0.347
**Severity scores**					
APACHE II	13±6	15±6	11±6		**0.004**
SAPS II	30±13	33±11	28±13		**0.045**
**Clinical features**					
GCS	15(15,15)	15(15,15)	15(15,15)		0.800
Respiratory rate	37±10	38±2	36±10		0.228
SpO _2_	85±11	85±6	84±12		0.434
Heart rate	97±23	107±27	92±20		**0.002**
MAP	96±15	100±15	94±15		**0.047**
**Initial laboratory finding**					
P/F ratio	122± 61	109±42	126±67		0.088
Baseline serum urea (g/L)	0.42±0.12	0.47±0.08	0.40±0.12		**< 0.001**
Baseline creatinine (μmol/L)	72±26	88±24	66±23		**< 0.001**
WBC count (×10 ^9^/L)	12±6	14±9	11±5		**0.024**
lymphocyte (×10 ^3^/μl)	1272±960	1304±1053	1260±926		0.817
Platelet (×10 ^9^/L)	277±130	290±134	271±128		0.450
CRP (mg/L)	154±97	181±87	143±98		**0.025**
Prothrombin time (%)	79±20	75±22	80±18		0.232
**CT scan lesion extension** (%)	60±20	63±19	58±19		0.317
**Respiratory support**					
HFNC	6 (4)	0 (0)	6 (6)		**< 0.001**
Alternation NIV/HFNC	54 (38)	6 (15)	48 (47)		**< 0.001**
MV	84 (58)	35 (85)	49 (48)		**< 0.001**
**Predisposing condition**					
Sepsis	61 (42)	22 (54)	39 (38)		0.083
shock	70 (49)	33 (80)	37 (36)	**2.21 (1.64-2.99)**	**< 0.001**
Rhabdomyolysis	13 (9)	2 (5)	11(11)		0.122
Nephrotoxic antibiotics	18 (12)	12 (29)	6 (6)	**5.02 (2.02-12.49)**	**< 0.001**
Iodine contrast agent	32 (22)	5 (12)	27 (26)		0.068
MV before AKI	79 (55)	30(73)	49(48)	**1.5 (1.18-2.07)**	**0.004**
**ICU LOS**	13±11	14±13	12±11		0.454
**Mortality rate**	93(65)	36 (88)	57 (55)		**< 0.001**

Values are presented as mean± SD (standard deviation) median (Q1, Q3) or number (%).

**BMI**: body mass index; **APACHE**: Acute Physiology and Chronic Health Evaluation; **SAPS**: Simplified Acute Physiology Score; **GCS**: Glasgow Coma Scale; **SpO _2_**: pulse oximeter oxygen saturation; **MAP**: mean arterial pressure; **P/F ratio**: ratio of the arterial partial pressure of oxygen and the inspiratory concentration of oxygen; **WBC**: white blood cell; **CRP**: C-reactive protein; **CT**: computed tomography; **HFNC**: high-flow nasal cannula; **NIV**: noninvasive ventilation; **MV**: mechanical ventilation; **ICU** intensive care unit; **LOS**: length of stay; **AKI**: acute kidney injury.

### Risk factors of AKI

According to the KDIGO criteria the AKI patients (41 cases) were staged class 1 (5 cases: 12%), class 2 (16 cases: 39%) or class 3 (20 cases: 49%). AKI occurred within a median of 4 days (3, 8.5) and extremes between 1 and 32 days. The mean creatinine level at the onset of the AKI was 285±185 μmol/L (extremes between 106 and 955 μmol/L). Thirteen patients (32%) underwent RRT. Renal function improved in seven cases (17%). As shown in Table 1: age, diabetes, hypertension, APACHE II, SAPS II, heart rate, MAP, serum baseline urea and creatinine, WBC count, CRP, shock, MV and nephrotoxic antibiotics were all predictors of AKI in univariate analysis. However, diabetes, nephrotoxic antibiotics, and shock were the three independent risk factors in the multivariate analysis ( Table 2).

**Table 2.** Independent risk factors of AKI onset.

Risk factors	Adjusted OR (95% CI)	P-value
**Diabetes**	6.07 (1.30-28.4)	0.022
**Nephrotoxic antibiotics**	21.68 (3.2-146)	0.002
**Shock**	12.21 (2.87-51.85)	0.031

### Outcomes

Mean ICU length of stay (LOS) was longer in AKI patients without a significant difference (p: 0.454) but mortality was significantly higher (88% versus 55%, p< 10 ^−3^) ( Table 1). Only five patients of the AKI group survived (three were classified KDIGO 1 and two KDIGO 2). All AKI KDIGO 3 patients had fatal outcome.

**Figure 2.**
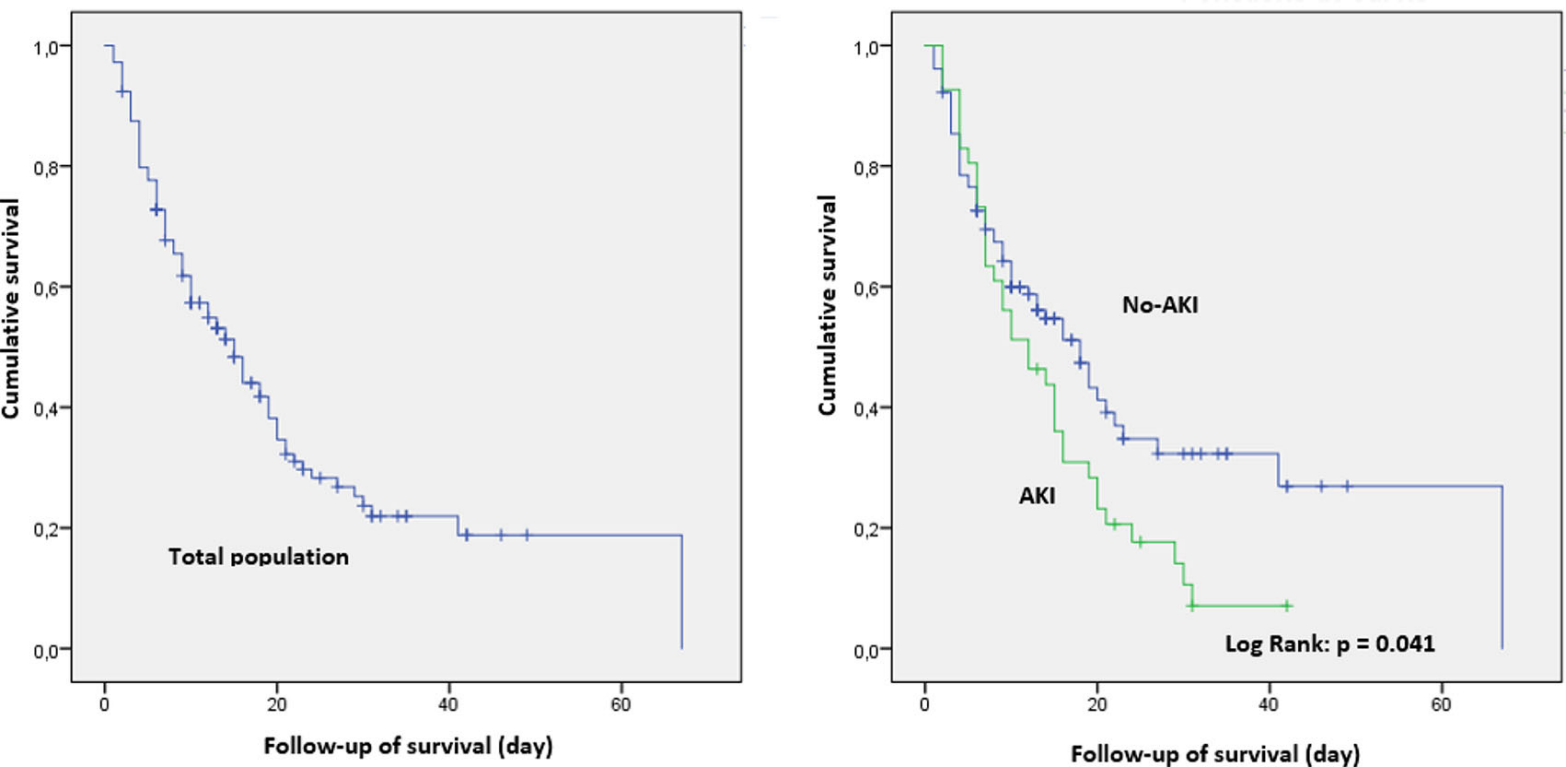
Survival curve of the total population and according to the occurrence of AKI.

Figure 2 shows the cumulative survival rates of the total population and the two groups according to the occurrence of AKI.

In univariate analysis, age > 53 years, severity scores, CT scan lesion extension > 67.5%, septic shock complicating hospital acquired infection (HAI), AKI, MV were all predictive of poor outcome. Besides, age > 53, septic shock complicating HAI and MV were the three independent factors of mortality ( Table 3).

**Table 3.** Factors associated with mortality.

Prognostic factors	Deceased n=93	Survivor n=51	Univariate analysis		Multivariate analysis	
			HR (95% CI)	P- value	aHR (95% CI)	P-value
**Age > 53 (years)**	76 (82)	22 (43)	**10 (6.69-13.3)**	**< 0.001**	** 1.45 (1.23-1.87)**	**0.018**
**CT scan lesion** **extension > 67.5 %**	38 (41)	13 (25)	**12 (7.25-16.74)**	**0.027**	-	0.247
**APACHE II > 14.5**	43 (46)	3 (6)	**6 (3.85-8.14)**	**< 0.001**	-	0.120
**SAPS II > 27.5**	60 (64)	12 (23)	**9 (6.57-11.42)**	**< 0.001**	-	0.923
**AKI**	36 (39)	5 (10)	**12 (5.81-18.18)**	**0.041**	-	0.641
**MV**	82 (88)	2 (4)	**9 (7.11-10.88)**	**< 0.001**	**8.20 (3.64-18.46)**	**< 0.001**
**Septic shock complicating HAI**	47 (50)	10 (20)	**16 (11.7-20.4)**	**0.003**	**2.43 (1.35-4.40)**	**0.003**

Values are presented as number (%).

**HR:** hazard ratio, **aHR:** adjusted HR, **AKI:** acute kidney injury, **MV:** mechanical ventilation **, HAI:** Hospital-acquired infection.

## Discussion

### Key results

In this study among the 144 patients enrolled, 41 (28%) developed AKI during ICU-hospitalization within a median of 4 days (3, 8.5). It was staged KDIGO 3 in about half of the cases. Thirteen patients underwent RRT and renal function improved in only seven cases. Diabetes, nephrotoxic antibiotics and shock were the three independent risk factors of AKI. Mortality was significantly higher in AKI group, but AKI didn’t appear to be an independent risk factor of poor outcome in multivariate analysis.

### Incidence of AKI in critical COVID-19 forms

In patients undergoing conventional hospitalization, the incidence of AKI ranged from 0.5% to 5,3%. ^3^^,^^16^^–^^18^

The prevalence of AKI increases in parallel with the COVID-19 severity. In the study by Hu et al, AKI occurred in 1.3% (2 of 151), 3.4% (5 of 146), and 38.5% (10 of 26) of non-severe patients, severe, and critical patients respectively. ^19^ Similar findings were reported by Zheng et al, who found an incidence of AKI of 1.0% (3 of 297), 6.8% (13 of 190), and 39.4% (13 of 33) in non-severe, severe, and critical patients, respectively. ^20^ In a systematic review and meta-analysis of 58 studies focused in AKI and RRT in COVID-19 patients, 13 studies reported on AKI incidence among critical patients. Overall, AKI occurred in 312/565 ICU patients with a pooled incidence rate of 39.0%. ^21^

There is also a difference in the prevalence of AKI depending on the patients’ geographical distribution. Data from Chinese studies estimated the AKI prevalence between 8.3% and 50.6% in ICU COVID-19 patients. ^16^^,^^22^^–^^25^ More recent studies, from the United States, have found a higher prevalence ranging from 19% to 76%. ^26^^–^^29^ A total of 61/215 (28.4%) patients admitted to a Sub-Saharan African ICU developed AKI. ^30^ This rate seems to be more important in European ICUs reaching levels above 80%. ^4^^,^^5^

AKI is also variable in severity. KDIGO is the most commonly used classification, and the kidney damage was staged KDIGO 1, 2 and 3 in 25-39%; 3.5-35% and 30-63% respectively in several previous series. ^25^^,^^27^^,^^28^^,^^30^ AKI is usually diagnosed within 5 to 9 days of hospital admission and a median of 12 to 21 days after the onset of symptoms. ^23^^,^^25^^,^^31^ However, Hirsch et al. reported a high frequency of AKI occurrence (37%) within 24 hours of admission. ^28^ Depending on the study, the use of RRT in ICU is variable from 16% to 73% of patients with AKI. ^4^^,^^5^^,^^16^^,^^23^^,^^25^^,^^28^^–^^30^

These discrepancies between studies concerning the incidence of AKI, its severity, its time of onset and the use of RRT could be explained by: variation of the definition of “severe” disease and AKI, heterogeneity of the studied populations, genetic predisposition to kidney involvement and RRT resource limitations.

The incidence of AKI was 28% in our study, which is a low rate compared to previous series. This may be explained by the fact that all patients included didn’t have a history of CKD. Moreover, as this population had critical clinical presentation with several AKI risk factors, AKI was rather severe (only 12% were classified KDIGO 1).

### Risk factors

**-Demographic risk factors**

In our study AKI patients were older than no AKI ones with a significant difference in univariate analysis, however age was not considered as an independent predictor of AKI in multivariate analysis. Older age was considered as a risk factor for AKI and RRT in an Italian cohort of 99 invasively ventilated COVID-19 patients. ^32^ Likewise, in a large Chinese study by Hirsh et al including 5449 COVID-19 patients, 1993 (36.6%) developed AKI and older age was an independent predictor of AKI (OR: 1.03; 95% CI: (1.03–1.04); p<0.001). ^28^ Similar findings were reported by Dereli et al. ^2^

Lin et al analyzed the data of 79 research articles: 8 studies investigated the risk factors of COVID-19 induced AKI and also showed that age ≥ 60 years and severe infection were independent factors predicting AKI with ORs: 3.53 (95% CI: (2.92-4.5); p<0.001), and 6.07 (95% CI (2.53-14.58); p<0.001) respectively. ^33^

While male gender was much more associated with AKI, as reported by Hirsh JS et al. ^28^ and Ng JH et al., ^34^ sex ratio was comparable in our cohort and other previous studies. ^2^^,^^4^^,^^32^

**-Comorbidities**

Most of the critical COVID-19 patients have pre-existing comorbidities which were also associated to AKI. The most common are hypertension and other cardiovascular disorders, diabetes and obesity. Diabetes was an independent factor in our study as well as in several series. ^28^^,^^34^ Hypertension was also significantly much more frequent in AKI patients in our study as well as in previous studies. ^2^^,^^28^ In addition, cardiomyopathy, chronic respiratory failure and BMI were also reported as risk factors of AKI. ^2^^,^^28^ According to these findings, in a recent meta-analysis of forty-four studies with a total number of 114 COVID-19 patients with AKI, Sabaghian et al found that factors including older age, hypertension, cardiovascular disease, diabetes, high BMI, chronic kidney disease, immunosuppression, and smoking are the potential risk factors of AKI. ^7^

These comorbidities are well-known factors of renal vulnerability causing histological lesions of nephroangiosclerosis or diabetic glomerulosclerosis which are considered as underlying renal fragility factors in COVID-19 patients. ^35^^–^^38^ Moreover, due to these conditions, patients are frequently treated with drugs that interfere with regulation of renal flow, such as ACE inhibitors. ^9^ Besides, AKI patients had higher baseline serum creatinine with a significant difference in our cohort and similar findings were reported in several studies. ^17^^,^^28^^,^^34^^,^^39^ This could be explained by the premorbid kidney disease potentially related to the frequent comorbidities especially diabetes and hypertension.

**-Acute disease severity and therapeutics**

In addition to these non-modifiable demographic factors, the severity of the COVID-19 on admission was the major predictor of AKI. In fact, severity scores were significantly higher in the AKI patients in our study and in several previous series. ^2^^,^^4^ In addition, ARDS requiring MV, shock and vasopressor support were reported as predictive of AKI. ^2^^,^^4^^,^^28^^,^^40^^,^^41^

Since AKI patients had more serious forms of COVID-19, they require much more MV which was predictive of AKI in our univariate analysis but was not considered an independent factor. In fact, critical COVID-19 patients are at a high risk of AKI as a complication of MV. Specifically, high positive end-expiratory pressure used for COVID-19 associated ARDS leads to increased intrathoracic pressure and can ultimately result in increased renal venous pressure and reduced filtration. ^42^ Besides, positive pressure ventilation can increase sympathetic tone, leading to secondary activation of the renin–angiotensin system. ^43^ Furthermore, upregulation of proinflammatory mediators associated to biotrauma, may subsequently induce multiple system organ failure including the kidney. the kidney-lung crosstalk theory is due to the increased release of cytokines in the blood, which is promoted by lung injury. Elevated levels of cytokines, especially IL-6, increase alveolar-capillary permeability and pulmonary hemorrhage. It even may lead to distant organs dysfunction, notably damage of the kidney vascular endothelium. ^44^

Moreover, restrictive fluid strategy recommended for ARDS patients, who may initially present with relative volume depletion due to fever and gastrointestinal losses, may worsen hypovolemia and compromise renal perfusion. ^45^ Thus, hypovolemia and hemodynamic instability cause renal hypoperfusion and, consequently, AKI. Moreover, shock is associated to lactic acidosis, hyperkalemia and rhabdomyolysis which all had a negative impact on kidney function. ^45^ Therefore, careful attention to volume status is needed to avoid AKI.

Beyond shock and diabetes, nephrotoxic antibiotics use was also an independent factor of AKI in our study. In fact, critical COVID-19 patients might be exposed to nephrotoxins as part of their clinical care, in particular, antibiotics, which can result in tubular injury or acute interstitial nephritis.

In a large Chinese study including 210 ICU COVID-19 patients, Sang et al proved that the use of nephrotoxic drug was an independent factor of AKI (OR: 2.67; 95% CI: (1.09–6.55); p: 0.0316). ^46^

Similarly, a Portuguese study including 192 COVID-19 patients (20% of whom needed ICU management), confirmed that the exposure to nephrotoxins during the first week of admission (vancomycin, aminoglycosides, nonsteroidal anti-inflammatory drug and iodine contrast agents) was an independent factor of AKI (OR 3.60 95% CI (1.30–9.94) p=0.014). ^39^

In a most recent study carried in Argentina including 162 ICU COVID-19 patients, exposure to nephrotoxic drugs (particularly polymyxins and aminoglycosides) was markedly higher in the AKI group (p<0.001). ^40^

The use of iodine contrast agents was not considered as an AKI risk factor in our cohort. This could be explained by the fact that, on the one hand, all patients included didn’t have previous CKD and on the other, they received hydro-electrolytic supplements according to the daily fluid balance calculated by subtracting the total fluid output from the total intake.

### Outcomes

Mean ICU LOS was longer in AKI patients without a significant difference but mortality rate was significantly higher in this group and all patients staged KDIGO 3 deceased.

In univariate analysis AKI was a poor prognostic factor but only age >53 years, septic shock complicating HAI and MV were the three independent factors of mortality.

Mortality was also significantly higher in AKI patients in the most reported studies and it increases in parallel with the AKI severity. ^30^^,^^39^^–^^41^

In fact, Nlandu Y et al. found that the death rate of AKI patients was more than 2 time higher than all patients. Besides, this rate was more than 3 times higher, in patients requiring RRT than those classified AKI stage 1. Thus, AKI was an independent prognostic factor in this study (OR: 2.96; 95% CI (1.23-4.65); p: 0.013). ^30^

Likewise, AKI stage 3 (OR: 5.33; 95% CI (1.15-24.65); p: 0.0321) was independently associated with death in the study by Sang L et al. in addition to critical disease (OR: 69.16; 95% CI (5.86-815.79); p: 0.0008), older age (OR: 1.06; 95% CI (1.02-1.11); p:0.0035) and P/F < 150 (OR: 15.21; 95% CI (4.72-49.07); p<10 ^−3^). ^46^

Beyond older age (OR: 1.07; 95% CI (1.02–1.11); p: 0.004), lower Hb level (OR: 0.78; 95% CI (0.60–0.98); p: 0.035), persistent AKI (OR: 7.34; 95% CI (2.37–22.72); p: 0.001) and severe AKI (OR: 2.65 per increase in KDIGO stage; 95% CI (1.32–5.33); p: 0.006) were also considered independent factors of mortality in the study by Gameiro et al. ^39^

Thus, most of the studies agree on the negative prognostic impact of AKI on critical COVID-19 patients and this is not surprising. In fact, as AKI most often occurs in elderly patients with multiple comorbidities, severe forms of COVID-19, and requiring life-sustaining therapies (particularly MV and vasopressor therapy), they are expected to have a poor prognosis. Although, our results showed that AKI was associated to mortality in univariate analysis, it wasn’t considered as an independent factor in multivariate analysis. This could be due to the fact that some factors were mutually dependent as shock, MV and AKI.

This study is one of the few works that have focused on the AKI in critical forms of COVID-19 managed in the ICU with a large number of patients which represent its strength. Although some limitations must be noted. The retrospective design of our study was constrained due to the paucity of data on the previous treatments of patients enrolled, notably, prior use of angiotensin converting enzyme inhibitor or angiotensin II receptor blocker. In addition, some laboratory tests were lacking in our hospital such us ferritin and D-dimers. Thus, these missing data considered as a risk factor for AKI in several studies could not be evaluated in our patients.

## Conclusion

The incidence of AKI was high in this study and associated to an increased mortality. Diabetes, nephrotoxic antibiotics and shock contributed significantly to its occurrence. This emphasizes the importance of rationalizing the antibiotic prescription and avoiding nephrotoxic drugs whenever possible. In addition, a rapid and adequate management of these critical patients may reduce hemodynamic instability and consequent organs failure. furthermore, careful monitoring of renal function and early detection of AKI can help to prevent its progression to a more severe stage associated with a poor prognosis. We recommend further multicenter studies with larger samples and more detailed data in order to support our results.

## Data Availability

All data are available in Zenodo. https://doi.org/10.5281/zenodo.10865485. ^47^ These data include aim and methods of the study, contributors, all information about patients with respect of confidentiality and anonymity, STROBE checklist and the consent form. Data are available under the terms of the Creative Commons Attribution 4.0 International license (CC-BY 4.0). Zenodo: STROBE checklist for “Acute kidney injury complicating critical forms of COVID-19: risk factors and prognostic impact”, https://doi.org/10.5281/zenodo.10865485. ^47^

## References

[1] *Therapeutics and COVID-19: living guideline, 14 July 2022.* Geneva: World Health Organisation;2022. (WHO/2019-nCoV/therapeutics/2022.4). License: CC BY-NC-SA 3.0 IGO.

[2] Dereli N Babayigit M Menteş O : Are we aware of COVID-19-related acute kidney injury in intensive care units? *Eur. Rev. Me. Pharmacol. Sci.* 2022;26:1753–1760. 10.26355/eurrev_202203_28245 35302225

[3] Guan WJ Ni ZY Hu Y : Clinical characteristics of coronavirus disease 2019 in China. *N. Engl. J. Med.* 2020;382:1708–1720. 10.1056/NEJMoa2002032 32109013 PMC7092819

[4] Rubin S Orieux A Prevel R : Characterization of acute kidney injury in critically ill patients with severe coronavirus disease 2019. *Clin. Kidney J.* 2020;13:354–361. 10.1093/ckj/sfaa099 32695326 PMC7314187

[5] Joseph A Zafrani L Mabrouki A : Acute kidney injury in patients with SARS-CoV-2 infection. *Ann. Intensive Care.* 2020;10:117. 10.1186/s13613-020-00734-z 32880774 PMC7471244

[6] Zheng X Zhao Y Yang L : Acute Kidney Injury in COVID-19: The Chinese Experience. *Semin. Nephrol.* 2020;40(5):430–442. 10.1016/j.semnephrol.2020.09.001 33334457 PMC7473017

[7] Sabaghian T Kharazmi AB Ansari A : COVID-19 and Acute Kidney Injury: A Systematic Review. *Front. Med.* 2022;9:705908. 10.3389/fmed.2022.705908 35445048 PMC9014846

[8] Battle D Soler MJ Sparks MA : Acute kidney injury in COVID-19: emerging evidence of a distinct pathophysiology. *J. Am. Soc. Nephrol.* 2020;31(7):1380–1383. 10.1681/ASN.2020040419 32366514 PMC7350999

[9] Gabarre P Dumas G Dupont T : Acute kidney injury in critically ill patients with COVID-19. *Intensive Care Med.* 2020;46:1339–1348. 10.1007/s00134-020-06153-9 32533197 PMC7290076

[10] Singer M Deutschman CS Seymour CW : The third international consensus definitions for sepsis and septic shock (Sepsis-3). *JAMA.* 2016;315:801–810. 10.1001/jama.2016.0287 26903338 PMC4968574

[11] Kidney Disease: Improving Global Outcomes (KDIGO) Acute Kidney Injury Work Group. KDIGO Clinical Practice Guideline or Acute Kidney Injury. *Kidney Int.* 2012;2:1–138.

[12] Forni LG Darmon M Ostermann M : Renal recovery after acute kidney injury. *Intensive Care Med.* 2017;43(6):855–866. 10.1007/s00134-017-4809-x 28466146 PMC5487594

[13] Stahl K Rastelli E Schoser B : A systematic review on the definition of rhabdomyolysis. J. Neurol. 2020Apr;267(4):877–882. 10.1007/s00415-019-09185-4 30617905

[14] SFR e-Bulletin: La société d’Imagerie Thoracique propose un compte-rendu structuré de scanner thoracique pour les patients suspects de COVID-19 (Thoracic Imaging Society offers structured chest CT scan report for patients suspected of COVID-19). Reference Source Reference Source

[15] The jamovi project: *jamovi* (Version 2.3) [Computer Software]. 2023. Reference Source

[16] Wang D Hu B Hu C : Clinical Characteristics of 138 Hospitalized Patients With 2019 Novel Coronavirus–Infected Pneumonia in Wuhan, China. *JAMA.* 2020;323:1061–1069. 10.1001/jama.2020.1585 32031570 PMC7042881

[17] Cheng Y Luo R Wang K : Kidney disease is associated with in-hospital death of patients with COVID-19. *Kidney Int.* 2020;97:829–838. 10.1016/j.kint.2020.03.005 32247631 PMC7110296

[18] Cao M Zhang D Wang Y : Clinical Features of Patients Infected with the 2019 Novel Coronavirus (COVID-19) in Shanghai, China. *MedRxiv.* 2020.

[19] Hu L Chen S Fu Y : Risk factors associated with clinical outcomes in 323 COVID-19 hospitalized patients in Wuhan, China. *Clin. Infect. Dis.* 2020;71(16):2089–2098. 10.1093/cid/ciaa539 32361738 PMC7197620

[20] Zheng X Yang H Li X : Prevalence of kidney injury and associations with critical illness and death in patients with COVID-19. *Clin. J. Am. Soc. Nephrol.* 2020;15(11):1549–1556. 10.2215/CJN.04780420 32943396 PMC7646240

[21] Yang X Tian S Guo H : Acute kidney injury and renal replacement therapy in COVID-19 patients: A systematic review and meta-analysis. *Int. Immunopharmacol.* 2021;90:107159. 10.1016/j.intimp.2020.107159 33223467 PMC7608016

[22] Huang Y Yang R Xu Y : Clinical characteristics of 36 non-survivors with COVID-19 in Wuhan, China. medRxiv Infectious Diseases (except HIV/AIDS). 2020.

[23] Yang X Yu Y Xu J : Clinical course and outcomes of critically ill patients with SARS-CoV-2 pneumonia in Wuhan, China: a single-centered, retrospective, observational study. *Lancet Respir. Med.* 2020;8(5):475–481. 32105632 10.1016/S2213-2600(20)30079-5 PMC7102538

[24] Pei G Zhang Z Peng J : Renal Involvement and Early Prognosis in Patients with COVID-19 Pneumonia. *J. Am. Soc. Nephrol.* 2020;31:1157–1165. 10.1681/ASN.2020030276 32345702 PMC7269350

[25] Xia P Wen Y Duan Y : Clinicopathological Features and Outcomes of Acute Kidney Injury in Critically Ill COVID-19 with Prolonged Disease Course: A Retrospective Cohort. *J. Am. Soc. Nephrol.* 2020;8:475–481. 10.1681/ASN.2020040426 PMC7461691 32826326

[26] Arentz M Yim E Klaff L : Characteristics and Outcomes of 21 Critically Ill Patients With COVID-19 in Washington State. *JAMA.* 2020;323(16):1612–1614. 10.1001/jama.2020.4326 32191259 PMC7082763

[27] Robbins-Juarez SY Qian L King KL : Outcomes for Patients With COVID-19 and Acute Kidney Injury: A Systematic Review and Meta-Analysis. *Kidney Int. Rep.* 2020;5(8):1149–1160. 10.1016/j.ekir.2020.06.013 32775814 PMC7314696

[28] Hirsch JS Ng JH Ross DW : Acute kidney injury in patients hospitalized with COVID-19. *Kidney Int.* 2020;98:209–218. 10.1016/j.kint.2020.05.006 32416116 PMC7229463

[29] Mohamed MMB Lukitsch I Torres-Ortiz AE : Acute Kidney Injury Associated with Coronavirus Disease 2019 in Urban New Orleans. *Kidney360.* 2020;1(7):614–622. 10.34067/KID.0002652020 35372932 PMC8815549

[30] Nlandu Y Makulo JR Essig M : Factors associated with acute kidney injury (AKI) and mortality in COVID-19 patients in a Sub-Saharan African intensive care unit: a single-center prospective study. *Ren. Fail.* 2023;45(2):2263583. 10.1080/0886022X.2023.2263583 37870858 PMC11001370

[31] Piñeiro GJ Molina-Andujar A Hermida E : Severe acute kidney injury in critically ill COVID-19 patients. *J. Nephrol.* 2021;34(2):285–293. 10.1007/s40620-020-00918-7 33387345 PMC7776310

[32] Fominskiy EV Scandroglio AM Monti G : Prevalence, characteristics, risk factors, and outcomes of invasively ventilated COVID-19 patients with acute kidney injury and renal replacement therapy. *Blood Purif.* 2021;50(1):102–109. 10.1159/000508657 32659757 PMC7445373

[33] Lin L Wang X Ren J : Risk factors and prognosis for COVID-19-induced acute kidney injury: a meta-analysis. *BMJ Open.* 2020;10(11): e042573. 10.1136/bmjopen-2020-042573 33172950 10.1136/bmjopen-2020-042573 PMC7656886

[34] Ng JH Hirsch JS Hazzan A : Outcomes Among Patients Hospitalized With COVID-19 and Acute Kidney Injury. *Am. J. Kidney Dis.* 2021;77(2): 204–15.e1. 10.1053/j.ajkd.2020.09.002 32961245 PMC7833189

[35] Sharma P Uppal NN Wanchoo R : COVID-19-associated kidney injury: a case series of kidney biopsy findings. *J. Am. Soc. Nephrol.* 2020;31(9):1948–1958. 10.1681/ASN.2020050699 32660970 PMC7461689

[36] Golmai P Larsen CP DeVita MV : Histopathologic and ultrastructural findings in postmortem kidney biopsy material in 12 patients with AKI and COVID-19. *J. Am. Soc. Nephrol.* 2020;31(9):1944–1947. 10.1681/ASN.2020050683 32675304 PMC7461690

[37] Su H Yang M Wan C : Renal histopathological analysis of 26 postmortem findings of patients with COVID-19 in China. *Kidney Int.* 2020;98:219–227. 10.1016/j.kint.2020.04.003 32327202 PMC7194105

[38] Kudose S Batal I Santoriello D : Kidney Biopsy Findings in Patients with COVID-19. *J. Am. Soc. Nephrol.* 2020;31:1959–1968. 10.1681/ASN.2020060802 32680910 PMC7461665

[39] Gameiro J Fonseca JA Oliveira J : Acute kidney injury in hospitalized patients with COVID-19: A Portuguese cohort. *Nefrologia.* 2021;41(6):689–698. 10.1016/j.nefro.2021.04.002 36165158 PMC8800378

[40] Rolón NC Varela CF Ferraris A : characteristics of acute kidney injury in adult patients with severe covid-19. *Medicina (B Aires).* 2022;82(2):172–180. 35417379

[41] Lumlertgul N Pirondini L Cooney E : Acute kidney injury prevalence, progression and long-term outcomes in critically ill patients with COVID-19: a cohort study. *Ann. Intensive Care.* 2021;11:123. 10.1186/s13613-021-00914-5 34357478 PMC8343342

[42] Koyner JL Murray PT : Mechanical ventilation and lung-kidney interactions. *Clin. J. Am. Soc. Nephrol.* 2008;3(2):562–570. 10.2215/CJN.03090707 18256378 PMC6631081

[43] Dudoignon E Moreno N Deniau B : Activation of the renin- angiotensinaldosterone system is associated with acute kidney injury in COVID-19. *Anaesth. Crit. Care Pain Med.* 2020;39(4):453–455. 10.1016/j.accpm.2020.06.006 32565254 PMC7301818

[44] Joannidis M Forni LG Klein SJ : Lung-kidney interactions in critically ill patients: consensus report of the acute disease quality initiative (ADQI) 21 workgroup. *Intensive Care Med.* 2020;46(4):654–672. 10.1007/s00134-019-05869-7 31820034 PMC7103017

[45] Głowacka M Lipka S Młynarska E : Acute Kidney Injury in COVID-19. *Int. J. Mol. Sci.* 2021;22(15):8081. 10.3390/ijms22158081 34360866 PMC8347536

[46] Sang L Chen S Zheng X : The incidence, risk factors and prognosis of acute kidney injury in severe and critically ill patients with COVID-19 in mainland China: a retrospective study. *BMC Pulm. Med.* 2020;20(1):290. 10.1186/s12890-020-01305-5 33167955 PMC7649893

[47] Guissouma J Ben Ali H Allouche H : Dataset: Acute kidney injury complicating critical forms of COVID-19: risk factors and prognostic impact.[Data set]. *Zenodo.* 2024. 10.5281/zenodo.10865485 PMC11747297 39839732

