# Supplementary material for: Acute Kidney Injury Complicating Critical Forms of COVID-19: risk Factors and Prognostic Impact
Source: F1000Res. 2025 Jan 2;13:497. Originally published 2024 May 17. [Version 3] doi: 10.12688/f1000research.144105.3 (PMC11747297; doi:10.12688/f1000research.144105.3)
Supplement: Peer review history

# Reviewer report: Reviewer response for version 3

**DOI:** 10.5256/f1000research.176287.r355902

**Published:** 2025-01-27

**Authors:** Md. Safiullah Sarker

**Affiliations:** 1 Virology Laboratory, icddr,b, Dhaka, Bangladesh

The article titled *"Acute Kidney Injury Complicating Critical Forms of COVID-19: Risk Factors and Prognostic Impact"* by Guissouma et al. is a well-written piece. I am presenting my review based on the framework outlined below:

 1. Clarity and Accuracy of Presentation

 •    Evaluation: Partly.

 •    Feedback: The article is well-structured and discusses a critical topic effectively. However, certain sections, such as the statistical analysis and methodology, could benefit from clearer language and additional detail for better comprehension. Citations are appropriate and up to date. Minor grammatical corrections are needed to enhance clarity.

 2. Study Design and Technical Soundness

 •    Evaluation: Yes.

 •    Feedback: The study design—a retrospective case-control analysis—aligns with the research objectives and is sound. The chosen timeframe and inclusion criteria effectively capture the study population. However, the authors should provide further justification for excluding patients with chronic kidney disease (CKD).

 3. Methodology Details

 •    Evaluation: Partly.

 •    Feedback: While the methodology is detailed, specific information on handling potential biases (e.g., selection bias) is lacking. Additionally, the description of how nephrotoxic antibiotics were identified and categorized could be more precise.

 4. Statistical Analysis

 •    Evaluation: Yes.

 •    Feedback: The statistical methods used (e.g., logistic regression, Cox regression) are appropriate for analyzing risk factors and outcomes. The presentation of results in tables and survival curves is clear. Adding confidence intervals for all reported outcomes would enhance interpretability.

 5. Source Data Availability

 •    Evaluation: Yes.

 •    Feedback: The data is available through Zenodo, ensuring transparency and reproducibility. The authors have followed ethical guidelines appropriately.

 6. Support for Conclusions

 •    Evaluation: Yes.

 •    Feedback: The conclusions are logically derived from the results. However, the statement that AKI is not an independent risk factor for poor outcomes could benefit from additional discussion on the dependency of variables such as shock and mechanical ventilation.

**Overall Comments**

 Strengths:

 •    The study addresses a significant and understudied topic in a region with limited data.

 •    The analysis is comprehensive, and findings are presented in a structured manner.

 Weaknesses:

 •    Clarity in the methods and minor grammatical issues.

 •    Limited discussion on implications for practice, particularly preventive strategies for AKI in critically ill COVID-19 patients.

 Recommendations

 1.    Expand on the rationale for excluding CKD patients and its impact on generalizability.

 2.    Provide clearer descriptions of methodology, particularly regarding nephrotoxic antibiotic categorization.

 3.    Enhance clarity in the text with minor grammatical corrections.

 4.    Discuss implications for clinical practice, focusing on how findings could guide better management of critically ill patients.

 With these revisions, the article will significantly enhance its impact and readability.

 Here are the minor grammatical corrections needed for the article "Acute Kidney Injury Complicating Critical Forms of COVID-19: Risk Factors and Prognostic Impact":

**Grammatical Corrections**

 1.    Title:

 o    Capitalize "risk Factors" to ensure consistency with title case conventions:

 Correction: Risk Factors and Prognostic Impact.

 2.    Abstract:

 o    Replace "It was staged KDIGO class 3, in about half of the cases" with

 Correction: It was staged as KDIGO class 3 in about half of the cases.

 o    Change "This underlines the importance of rationalizing antibiotic prescription and providing adequate management" to

 Correction: This emphasizes the importance of rationalizing antibiotic prescriptions and providing adequate management.

 3.    Introduction:

 o    "Although, physicians must be aware of the possible damage of other organs causing a multi-systemic impairment."

 Correction: However, physicians must be aware of the potential damage to other organs, leading to multi-systemic impairment.

 o    "Acute kidney injury (AKI) is a frequent complication in COVID-19 patients with a reported incidence widely ranging from 0.5%3 to above 80%."

 Correction: Acute kidney injury (AKI) is a frequent complication in COVID-19 patients, with a reported incidence ranging widely from 0.5% to over 80%.

 4.    Methods:

 o    "Patients were divided in two groups"

 Correction: Patients were divided into two groups.

 o    "A rapid and adequate management of these critical patients may reduce hemodynamic instability and consequent organs failure."

 Correction: Rapid and adequate management of these critical patients may reduce hemodynamic instability and subsequent organ failure.

 5.    Results:

 o    "This rate seems to be more important in European ICUs reaching levels above 80%"

 Correction: This rate appears to be higher in European ICUs, reaching levels above 80%.

 o    "Thirteen patients underwent RRT and renal function improved in seven cases."

 Correction: Thirteen patients underwent RRT, and renal function improved in seven cases.

 6.    Discussion:

 o    "Mortality was significantly higher in AKI group, but AKI didn’t appear to be an independent risk factor of poor outcome in multivariate analysis."

 Correction: Mortality was significantly higher in the AKI group, but AKI did not appear to be an independent risk factor for poor outcomes in multivariate analysis.

 o    "This could be because some factors were mutually dependent as shock, MV, and AKI."

 Correction: This could be due to the fact that some factors, such as shock, MV, and AKI, were mutually dependent.

 7.    Conclusion:

 o    "Further multicenter studies with larger samples and more detailed data to support our results."

 Correction: Further multicenter studies with larger samples and more detailed data are needed to support our results

Is the work clearly and accurately presented and does it cite the current literature?

Partly

If applicable, is the statistical analysis and its interpretation appropriate?

Yes

Are all the source data underlying the results available to ensure full reproducibility?

Yes

Is the study design appropriate and is the work technically sound?

Yes

Are the conclusions drawn adequately supported by the results?

Yes

Are sufficient details of methods and analysis provided to allow replication by others?

Partly

Reviewer Expertise:

Virology

I confirm that I have read this submission and believe that I have an appropriate level of expertise to confirm that it is of an acceptable scientific standard, however I have significant reservations, as outlined above.

# Reviewer report: Reviewer response for version 3

**DOI:** 10.5256/f1000research.176287.r355402

**Published:** 2025-01-20

**Authors:** yannick Nlandu

**Affiliations:** 1 Nephrology Unit, Kinshasa University Hospital, Kinshasa, Democratic Republic of the Congo

I would like to thank the editor and the authors for the opportunity to review this very interesting manuscript. We feel that the authors have taken our comments on board and provided clear answers to our questions. The manuscript is now improved and ready for indexing.

Is the work clearly and accurately presented and does it cite the current literature?

Partly

If applicable, is the statistical analysis and its interpretation appropriate?

Partly

Are all the source data underlying the results available to ensure full reproducibility?

Partly

Is the study design appropriate and is the work technically sound?

Yes

Are the conclusions drawn adequately supported by the results?

Partly

Are sufficient details of methods and analysis provided to allow replication by others?

Partly

Reviewer Expertise:

AKI associated with COVID-19

I confirm that I have read this submission and believe that I have an appropriate level of expertise to confirm that it is of an acceptable scientific standard.

# Reviewer report: Reviewer response for version 2

**DOI:** 10.5256/f1000research.169953.r338920

**Published:** 2024-11-19

**Authors:** Nur Canpolat

**Affiliations:** 1 Cerrahpasa Faculty of Medicine, Department of Pediatric Nephrology, Istanbul University-Cerrahpasa, Istanbul, Turkey

The study reports that 28% of 144 adult COVID-19 patients developed AKI during ICU-hospitalization. The main risk factors for AKI were diabetes, the use of nephrotoxic drugs, and shock.

 Overall, the study is well-written and highlights significant issues. However, there are a few areas where the study could be further enhanced.

 The study does not provide details on pre-hospitalization treatments, especially the use of ACE inhibitors or angiotensin receptor blockers, which could influence kidney outcomes. If possible, including this information could may improve the analysis of the risk factors.

 The discussion section can be shorten reducing extra details to improve clarity.

 A minor but notable suggestion is to present p values as <0.001 rather than ****< 10 ^-3, ^which is more commonly used.

Is the work clearly and accurately presented and does it cite the current literature?

Yes

If applicable, is the statistical analysis and its interpretation appropriate?

Yes

Are all the source data underlying the results available to ensure full reproducibility?

Yes

Is the study design appropriate and is the work technically sound?

Yes

Are the conclusions drawn adequately supported by the results?

Yes

Are sufficient details of methods and analysis provided to allow replication by others?

Yes

Reviewer Expertise:

NA

I confirm that I have read this submission and believe that I have an appropriate level of expertise to confirm that it is of an acceptable scientific standard.

# Response

**Published:** 2024-12-22

**Authors:** Jihene Guissouma

**Affiliations:** medical intensive care department of Habib Bougatfa teaching hospital Bizerte Tunisia, Tunisia

**Dear reviewer:**

 Thanks for the time you have devoted to review this paper and for giving us the opportunity to benefit from your expertise. Here are the answers to your comments:    

 - We agree that details on pre-hospitalization treatments are lacking in our study. This was mentioned in the latest section in the article as “The retrospective design of our study was constrained due to the paucity of data on the previous treatments of patients enrolled, notably, prior use of angiotensin converting enzyme inhibitor or angiotensin”.                     

 - We have changed p values < 10 ^-3 ^with < 0.001 as recommended.

# Reviewer report: Reviewer response for version 2

**DOI:** 10.5256/f1000research.169953.r313362

**Published:** 2024-08-30

**Authors:** yannick Nlandu

**Affiliations:** 1 Nephrology Unit, Kinshasa University Hospital, Kinshasa, Democratic Republic of the Congo

I would like to thank the authors for giving me the opportunity to review the revised version of this very interesting manuscript on COVID associated with AKI.

 This very interesting manuscript has been improved after some changes made by the authors.  However, some issues remain unresolved.

 Methodology

 Statistical analysis

 - Logistic regression is not the best way to assess factors associated with mortality. Logistic regression is not the best way to assess factors associated with mortality. It is common to use Cox regression. Why didn't you use Cox regression in this retrospective study? If the authors could define a follow-up time from ICU admission to discharge, it would be really interesting to do Cox regression. 

 Outcome measures

 How can the author explain a GCS of 15 in the whole group, AKI and non-AKI groups with equal proportions (Table 1)?

 We have some concerns. How can the authors explain that with a high proportion of diabetic and hypertensive patients, an age of 58 ± 13 years and a baseline creatinine of 72 ± 26 ml/min, there were no patients with CKD?

Is the work clearly and accurately presented and does it cite the current literature?

Partly

If applicable, is the statistical analysis and its interpretation appropriate?

Partly

Are all the source data underlying the results available to ensure full reproducibility?

Partly

Is the study design appropriate and is the work technically sound?

Yes

Are the conclusions drawn adequately supported by the results?

Partly

Are sufficient details of methods and analysis provided to allow replication by others?

Partly

Reviewer Expertise:

AKI associated with COVID-19

I confirm that I have read this submission and believe that I have an appropriate level of expertise to confirm that it is of an acceptable scientific standard, however I have significant reservations, as outlined above.

# Response

**Published:** 2024-12-22

**Authors:** Jihene Guissouma

**Affiliations:** medical intensive care department of Habib Bougatfa teaching hospital Bizerte Tunisia, Tunisia

**Dear reviewer:**

 Thanks for the time you have devoted to review the revised version of this manuscript. Here are the answers to your comments:

**Methodology**

 - Statiscal analysis: We have used the receiver operating characteristic (ROC) curve to determine the cut-off values of the continuous data which were subsequently transformed into dichotomous form.  A comparison survival curve was obtained by means of the Log Rank test. Multivariable cox regression model was used to identify independent factors associated with mortality as recommended.

 As results, AKI was associated to mortality in univariate analysis but it wasn’t considered as an independent factor of mortality in multivariate analysis. This could be explained as we stated in the article by the fact that some factors were mutually dependent as shock, MV and AKI.         

**Outcome measures**

 - For the GCS: patients hospitalized with critical forms of COVID 19 had pronounced arterial hypoxemia without proportional signs of respiratory distress which known as silent or happy hypoxemia. Despite being very hypoxic, they had a good level of consciousness. In our cohort, 123 patients (86%) had a GCS of 15/15 and only seven (4.9%) were comatose. This explain why we had medians and interquartile ranges for the GCS of 15/15 for the total population and the two groups.

 - For the high proportion of diabetic and hypertensive patients, an age of 58 ± 13 years and a baseline creatinine of 72 ± 26 ml/min without CKD: It is possible that some of these patients may have histological lesions of nephroangiosclerosis or diabetic glomerulosclerosis. However, no previous diagnoses of CKD were recorded in their medical histories.

# Reviewer report: Reviewer response for version 1

**DOI:** 10.5256/f1000research.157850.r280604

**Published:** 2024-06-05

**Authors:** yannick Nlandu

**Affiliations:** 1 Nephrology Unit, Kinshasa University Hospital, Kinshasa, Democratic Republic of the Congo

I would like to thank the authors for giving me the opportunity to review this interesting article on AKI-associated COVID.

 This manuscript is certainly interesting but needs some changes to improve it.

 Introduction

 Currently, several published studies focused on hospitalized patients with COVID-19 and AKI but data on AKI complicating critical forms of COVID-19 are scarce. In this study we aimed to assess the incidence, the risk factors and the prognostic impact of AKI complicating critical forms of COVID-19.

- We think the authors need to rephrase their objective, as we believe we have a considerable number of studies on AKI in critically ill COVID-19 patients. Maybe this rarity is regional in Africa? In the Maghreb? The authors need to improve the justification of their study. Are there specific features of the Tunisian population and health care that could explain the high or low incidence of AKI? Any particular risk factor?

Method

 The primary endpoint was the incidence of AKI complicating severe forms of COVID-19. The secondary endpoints were risk factors for acute kidney injury and prognostic factors.

- How should risk factors be defined as an endpoint? We believe that risk factors cannot be defined as an endpoint.
- The authors also need to be clear about the prognostic factors: length of stay in intensive care, vital status, etc.

All adult patients (>18 years) admitted to the ICU for critical forms of COVID-19 during the study period were included. Patients with a history of chronic renal failure and those who did not meet the critical COVID-19 criteria were excluded.

 In the inclusion or exclusion criteria, the authors should specify whether they only included patients with at least two creatinine measurements.

- Why did the authors exclude all patients with CKD and not just those with end-stage renal disease?
- Could the authors provide a flow chart of patient selection?

The control group, which included those who retained normal renal function: No patients with AKI.

- We believe it is correct to present patients without AKI as those who do not meet the KDIGO criterion for AKI. Normal renal function seems a bit of a misnomer.

Only sepsis prior to the development of AKI was assessed as a risk factor.

- The authors need to be more precise about the time between sepsis and AKI to be taken into account: 24 hours or 48 hours before?

- AKI was defined by the Kidney Disease: Improving Global Outcomes (KDIGO) as any of the following

- Please state whether all patients have baseline creatinine or how the authors defined baseline creatinine in their study.
- Please provide a definition of AKI recovery as the authors reported an incidence of improved renal function.

Rhabdomyolysis was retained if the creatine phosphokinase (CPK) level was greater than five times the upper limit of normal.

- Could you please provide the reference for this definition? And please indicate the normal range of CPK used in the study.

We focused for each patient on demographic and clinical features, initial laboratory findings, initial thoracic computed tomography (CT) scan data, drugs received prior to AKI onset, respiratory support, renal function during hospitalization, need for RRT, ICU length of stay (LOS) and mortality

- The authors should be more precise in describing the data. For example, what clinical or biological data were collected? Age? Gender? Ethnic origin? CRP? Procalcitonin?
- How was the percentage of lesions on CT assessed? According to which classification?

Statistical analysis

- The authors should specify how the data were collected and analysed (Excel? Stata? SPSS?) Multivariate logistic regression analysis was performed to assess risk factors for AKI and those associated with ICU mortality.
- Logistic regression is not the best way to assess factors associated with mortality. Logistic regression is not the best way to assess factors associated with mortality. It is usual to use Cox regression. Why didn't you use a Cox regression model for this retrospective study?

Results

 Renal function improved in seven cases (17%)

 Please provide in the method section the definition of AKI recovery

 How can the author explain a GCS of 14±2?

 As in the result Ioded contrast was analyzed separately, we suggest to the authors to do the same in the method section.

 We have some concern. How the authors could explain the fact that with a high proportion of diabetic and hypertensive patients, an age of 58 years there is no CKD patients?

 Discussion

- We believe that the discussion section is too long and should be rewritten. The authors should only comment on the relevant results of the study. The authors should discuss the incidence of AKI and the AKI rates, which seem too low. The authors should discuss the risk factors reported in their results: diabetes, nephrotoxic antibiotics and shock. The authors should comment on the lack of association between iodinated contrast agent and AKI, which we believe is of great interest in reducing negative ideas about these agents. In this case, authors should specify in the methods section which type of iodinated contrast agent was used in their hospital.
- The authors should comment on mortality and the fact that they did not find AKI to be a risk factor for mortality in their cohort.

The clinical presentation of COVID-19 is variable, ranging from asymptomatic forms to severe ARDS. Common symptoms of COVID-19 include fever, fatigue, dry cough, and muscle ache, however critical forms may progress to ARDS, septic shock, multi organ failure and death. ^13^

_We think that it is not important_ _to add this_

 Thus, disease severity was classified into three categories: Critical COVID-19 is the most serious form. ^1^ Initially, physicians were focused in the respiratory manifestations but it was realized that multiple other organs could be also damaged. Multiple organ involvement including the liver, the gastrointestinal tract and/or the kidneys was described during the course of SARS in 2003 ^14^ and more recently in the early Chinese publications on COVID-19. ^15^

 Current literature suggests that kidney represents a potential target for SARS-COV 2: AKI is the most frequent manifestation. Data from first Chinese studies seemed to suggest a high incidence of renal complications among hospitalized patients. In patients undergoing conventional hospitalization, the incidence of AKI ranged from 0.5% to 5,3%

 Idem to be deleted

 The authors found a low number of stage 1 AKI cases in their cohort. We believe that the authors could add a possible explanation to these results.

 Pathophysiology of AKI

 We think that the design and the results of the study don’t allow to the authors to discuss pathophysiology of COVID-AKI

 they require mush more MV 

 Please correct mush to much

 that he use of nephrotoxic drug was an

 Please correct the use

 We think that the authors can use the reference of this African study where many results are similar such as the incidence of AKI, the incidence of AKI recovery, the Severity score, the aminoglycoside... as risk factors for AKI.

- Nlandu Y, et. al., 2023 (Ref 1) .

Is the work clearly and accurately presented and does it cite the current literature?

Partly

If applicable, is the statistical analysis and its interpretation appropriate?

Partly

Are all the source data underlying the results available to ensure full reproducibility?

Partly

Is the study design appropriate and is the work technically sound?

Yes

Are the conclusions drawn adequately supported by the results?

Partly

Are sufficient details of methods and analysis provided to allow replication by others?

Partly

Reviewer Expertise:

AKI associated with COVID-19

I confirm that I have read this submission and believe that I have an appropriate level of expertise to confirm that it is of an acceptable scientific standard, however I have significant reservations, as outlined above.

## References

[1] : Factors associated with acute kidney injury (AKI) and mortality in COVID-19 patients in a Sub-Saharan African intensive care unit: a single-center prospective study. *Ren Fail* .2023;45(2) : 10.1080/0886022X.2023.2263583 2263583 10.1080/0886022X.2023.2263583 37870858 PMC11001370

# Response

**Published:** 2024-08-02

**Authors:** Jihene Guissouma

**Affiliations:** medical intensive care department of Habib Bougatfa teaching hospital Bizerte Tunisia, Tunisia

**Dear Reviewer:**

 Thanks for the time you have devoted to review this paper and for giving us the opportunity to benefit from your expertise. Here are the answers to your constructive comments :

 In fact, studies about AKI in critically ill patients from the great Maghreb and particularly from Tunisia are rare (to our knowledge 3 research articles and one case report). AKI was a common evolutionary complication in patients hospitalized in our ICU. That’s why we were interested to carry out this study (we have clarified this point in version 2).

**Methods**

 -You are absolutely right. Risk factors cannot be defined as end points and for prognostic factors we mean vital prognosis (we have corrected this point in version 2).

 -For inclusion criteria we specified that all patients included had at least one creatinine measurement in ICU and one or more prior measurement in the department from which they were transferred or in the 7-365 days before admission.

 -All patients with CKD were excluded to have a homogeneous group of patients and avoid confounding factors.

 - A flow chart of patient selection was added to version 2.

 -For the two groups we corrected this point as: Patients were divided in two groups: the case group which included the critical COVID-19 patients who developed AKI during their ICU stay according to the Improving Global Outcomes (KDIGO) classification: AKI patients, and the control group which included those who didn’t develop AKI during their ICU stay according to the same classification: No AKI patients.

 -For sepsis we clarified this point in the new version: AKI was related to sepsis when a new episode of sepsis occurred during hospitalization and was followed within 48 hours by AKI. Sepsis was considered as AKI risk factor when no other cause of renal failure has been found.

 -For the baseline creatinine: for patients who had previous serum creatinine in the 7–365 days prior to admission, the most recent serum creatinine value was taken as the baseline creatinine and for whom no prior value was available, the lowest creatinine measured in the original department before transfer to ICU was considered baseline serum creatinine

 -Definition of renal recovery: Full renal recovery was achieved when serum creatinine reached a value below 1.5 times baseline and urine volume > 0.5 ml/Kg/h.   (Forni LG, Darmon M, Ostermann M, et al. Renal recovery after acute kidney injury. Intensive Care Med. 2017;43(6):855–866.)

 -For the definition of rhabdomyolysis we clarified in the new version that we referred to this systematic review: Chavez LO, Leon M, Einav S, Varon J. Beyond muscle destruction: a systematic review of rhabdomyolysis for clinical practice. Crit Care. 2016 Jun 15;20(1):135. doi: 10.1186/s13054-016-1314-5. PMID: 27301374; PMCID: PMC4908773.

 -Normal CPK rates range from 10 to 200 UI/l according to our hospital laboratory.

 -For data collection we have specified that: we focused for each patient on demographic (age and gender) and clinical features (comorbidities, data from pleuropulmonary, cardiovascular and neurological examinations) initial laboratory findings (arterial blood gases, renal function tests, complete blood count, CRP levels, prothrombin time, CPK), initial thoracic computed tomography (CT) scan data, drugs received prior to AKI onset, respiratory support, renal function during hospitalization, need for RRT, ICU length of stay (LOS) and mortality.

 -The classification used in our study is that of the French “Société d'Imagerie Thoracique”, based on a visual assessment of parenchymal extension into 5 stages according to the percentage of lung affected: absent or minimal involvement (< 10%), moderate (10-25%), extensive (25-50%), severe (50-75%) or critical (>75%).

 https://ebulletin.radiologie.fr/actualites-covid-19/societe-dimagerie-thoracique-propose-compte-rendu-structure-scanner-thoracique

**Statistical analysis**

 As indicated in the article, we used Jamovi software for data collection and analysis. Perhaps we should have used the Cox model, but multivariate analysis was chosen because it's the tool we're most proficient with.

**Results**

 -The definition of AKI recovery was clarified in the methods section.

 -For the GCS of 14±2: you are right, it was an oversight on our part: in fact this variable had a non-Gaussian distribution and we should have used the median and interquartiles we have corrected this in the new version.

 -For the high proportion of diabetic and hypertensive patients, an age of 58 years without CKD: perhaps, those patients had histological lesions of nephroangiosclerosis or diabetic glomerulosclerosis but not yet at a stage of CKD. These histological lesions are considered as underlying renal fragility factors in COVID-19 patients developing AKI.

**Discussion**

 We rewrote the discussion according to your recommendations and the sections that you suggest not important were deleted. Thus, we addressed that:

 -The low incidence of AKI in our study compared to previous series may be explained by the fact that all patients included didn’t have a history of CKD.

 -Omnipaque 300 was the iodinated contrast agent used in our Hospital. the lack of association between iodinated contrast agent and AKI in this study could be explained by the fact that, on the one hand, all patients didn’t have previous CKD and on the other, they received hydro-electrolytic supplements according to the daily fluid balance calculated by subtracting the total fluid output from the total intake.

 -Although, AKI was associated to mortality in univariate analysis it wasn’t considered as independent factor in multivariate analysis. This could be due to the fact that some factors were mutually dependent as shock, MV and AKI.

 -For the low number of stage 1 AKI cases in this cohort: we think that AKI was rather severe (only 12% were classified KDIGO 1) because this population had critical clinical presentation with several AKI risk factors.

 -Thank you for suggesting the reference of the African study we used it in different sections in the new version.

 We hope that we have replied to all your queries and are available if any other points remain unclear.

**Best regards**
